# Chromosome-level genome assembly of an agricultural pest *Zeugodacus tau* (Diptera: Tephritidae)

**DOI:** 10.1038/s41597-023-02765-0

**Published:** 2023-12-01

**Authors:** Yi-Ting Wang, Li-Jun Cao, Jin-Cui Chen, Wei Song, Wei-Hua Ma, Jing-Fang Yang, Xu-Yuan Gao, Hong-Song Chen, Yan Zhang, Zhen-Ya Tian, Shu-Jun Wei, Zhong-Shi Zhou

**Affiliations:** 1 State Key Laboratory for Biology of Plant Diseases and Insect Pests, Institute of Plant Protection, Chinese Academy of Agricultural Sciences, Beijing, 100193 China; 2 Institute of Plant Protection, Beijing Academy of Agriculture and Forestry Sciences, Beijing, 100097 China; 3 National Nanfan Research Institute, Chinese Academy of Agricultural Sciences, Sanya, 572019 China; 4 Hubei Insect Resources Utilization and Sustainable Pest Management Key Laboratory, College of Plant Science and Technology, Huazhong Agricultural University, Wuhan, 430070 China; 5 Guangxi Key Laboratory for Biology of Crop Diseases and Insect Pests, Institute of Plant Protection, Guangxi Academy of Agricultural Sciences, Nanning, 530007 China

**Keywords:** Entomology, Genome

## Abstract

The fruit fly *Zeugodacus tau* (Diptera: Tephritidae) is a major pest of melons and other cucurbits in Southeast Asia. In this study, we used Illumina, Nanopore, and Hi-C sequencing technologies to assemble a reference genome of *Z. tau* at the chromosomal level. The assembled genome was 421.79 Mb and consisted of six chromosomes (one X-chromosome + five autosomes). The contig N50 was 4.23 Mb. We identified 20,922 protein-coding genes, of which 17,251 (82.45%) were functionally annotated. Additionally, we found 247 rRNAs, 435 tRNAs, 67 small nuclear RNAs, and 829 small RNAs in the genome. Repetitive elements accounted for 55.30 Mb (13.15%) of the genome. This high-quality genome assembly is valuable for evolutionary and genetic studies of *Z. tau* and its relative species.

## Background & Summary

The tau fruit fly *Zeugodacus tau* (Diptera: Tephritidae) is a polyphagous pest that has invaded many regions worldwide, causing serious agricultural losses^1^. This species was previously classified in the subgenus *Zeugodacus* of the genus *Bactrocera*. Recently, the subgenus *Zeugodacus* was elevated to the genus level^2^. Species of *Zeugodacus* are considered more harmful than those of *Bactrocera* due to their high adaptability and invasive ability^3^. *Zeugodacus tau* has been listed as a quarantine species in many regions and countries, including China, the United States, Indonesia, Pakistan, and Japan^4,5^. Currently, *Z. tau* is distributed in most regions of southern China. It is generally present in tropical and subtropical Asia, sub-equatorial Africa, Australia, the Solomon Islands, and the South Pacific region^3,6^. Field monitoring has shown that *Z. tau* continues to expand to the high-latitude areas. However, there is limited data on historical records, and this species’ origin and colonization history remain unknown. Genetic studies may help reveal the adaptation and predict the future dispersal of this species. Due to the lack of genome data, studies on the invasion and genetics of *Z. tau* have been limited to the mitochondrial level^7^. Obtaining genomic data for this worldwide invasive insect could aid in controlling the spread of this pest and provide information on other invasive species.

In this study, we assembled a chromosome-level genome of *Z. tau* using a combination of Nanopore long-read, Illumina short-read sequencing, and chromosome conformation capture (Hi-C) technologies. We then performed structural and functional annotation on the obtained genome, incorporating transcriptome data from all developmental stages of *Z. tau*. This high-quality reference genome of *Z. tau* serves as a valuable resource for understanding the genetics, ecology, and evolution of *Z. tau* and providing information on the environmental adaptability and invasion mechanism of Tephritidae pests.

## Methods

### Sample preparation and genomic DNA sequencing

*Zeugodacus tau* samples were collected from Guangxi, China. They were reared for approximately nine generations in the laboratory under the following conditions: temperature of 27 ± 1 °C, relative humidity of 65 ± 5%, and a photoperiod of 14 L:10D. For genome sequencing, one pupa with unknown sex was used for the Nanopore library and Illumina library. Genomic DNA was extracted using the CTAB method and purified using a Blood and Cell Culture DNA Midi Kit (QIAGEN, Germany). The purity of the extracted DNA was determined using 0.75% agarose gel electrophoresis, and the concentration was assessed using a Qubit 2.0 Fluorometer from Thermo Fisher Scientific, USA. An Illumina paired-end (PE) library was constructed with an insert size of approximately 350 bp using the TruSeq Nano DNA HT Sample Preparation Kit (Illumina, San Diego, California, USA). The library was sequenced on the Illumina NovaSeq 6000 platform to generate paired-end reads of 150 bp. A total of 24.18 Gb (57.33 × coverage) of clean data was generated (Table 1). A long-insert library was also constructed using the same genomic DNA but with the SQK-LSK108 1D Ligation Sequencing Kit (Oxford Nanopore Technologies, Kidlington, Oxford, UK). This library was sequenced on the Nanopore PromethION sequencer at GrandOmics. The sequencing resulted in 51.67 Gb (122.50 × coverage) of long-reads, with an N50 length of 22,320 bp and an average length of 14,781.67 bp (Table 1).

**Table 1:** Library sequencing data and methods used in this study to assemble the *Zeugodacus tau* genome.

Sequencing strategy	Platform	Usage	Insertion size	Clean data (Gb)	Coverage (X)
Short-reads	Illumina	Genome survey	350 bp	24.18	57.33
Long-reads	Nanopore	Assembly	12–20 kb	51.67	122.50
Hi-C	Illumina	Hi-C assembly	350 bp	110.05	260.90
RNA-seq	Illumina	Annotation	350 bp	84.20	199.72

### Hi-C library preparation and sequencing

Two pupae with unknown sex were used to create the Hi-C library to capture genome-wide chromatin interactions. Chromatin digestion was carried out using the restriction enzyme MboI. The Hi-C samples were then extracted through biotin labeling, flat-end ligation, and DNA purification. The Hi-C library was sequenced using the Illumina NovaSeq platform with paired-end 150-bp reads. A total of 110.05 Gb (260.90 × coverage) of clean data were generated (Table 1).

### Transcriptome sequencing

For transcriptome sequencing, we collected three groups of samples. Each group consisted of five larvae, five pupae, five male and female adults, respectively, along with approximately 100 eggs. We extracted total RNA using the TRIzol reagent (Thermo Fisher Scientific, USA). Paired-end libraries were constructed using the VAHTSTM mRNA-seq V2 Library Prep Kit (Vazyme, Nanjing, China). The libraries were then sequenced on the Illumina NovaSeq 6000 platform with PE reads of 150 bp for subsequent genome annotation. A total of 84.20 Gb (199.72 × coverage) of clean data were generated (Table 1).

### Estimation of genomic characteristics

**Fig. 1:**
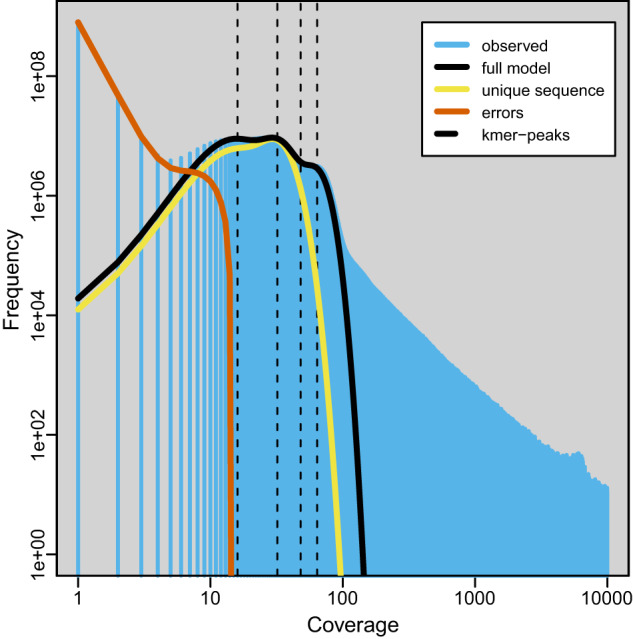
The estimated characteristics of *Zeugodacus tau* genome based on Illumina short-read data using 21-mers count histogram. Genome size was estimated to be 548.38 Mb, with a duplication rate of 1.12% and heterozygosity rate of 0.97%.

The K-mer method was utilized to survey the genome features of *Z. tau* with the Illumina short reads. The k-mer count histogram was calculated from Illumina short reads using Jellyfish^8^ version 2.2.10 with the parameters: ‘count -m 21 -C -s 5 G’. Genome size, heterozygosity, and duplication rate were estimated using GenomeScope^9^ version 1.0. The 21-mer analysis estimated the genome size of *Z. tau* to be approximately 548.38 Mb, with a high degree of duplication (1.12%) and heterozygosity (0.97%) (Fig. 1).

### Genome assembly

The Nanopore long reads were corrected and assembled into contigs using NextDenovo version 1.2.5 (https://github.com/Nextomics/NextDenovo) with parameters: ‘read_cutoff = 1k, genome_size = 400 m, pa_correction = 20, nextgraph_options = -a 1’. These contigs were then polished for three iterations using NextPolish version 1.4.0^10^ with the parameters: ‘genome_size = auto, sgs_options = -max_depth 100 -bwa, rerun = 3’. Subsequently, polished sequences were assembled into a chromosomal level based on Hi-C reads using Juicer version 1.6 with default parameters and 3D-DNA (3D *de novo* assembly, version 180922) pipelines^11–13^ with a modified parameter of ‘--editor-repeat-coverage 10’. The scaffolds were ordered manually using Juicebox version 1.11.08 (https://github.com/aidenlab/Juicebox) to obtain the final chromosome assembly. Syntenic blocks between chromosomes of *Z. tau* and *Drosophila melanogaster*^14^ were detected using MCScan^15^ based on the genome assembly and annotation results

**Fig. 2:**
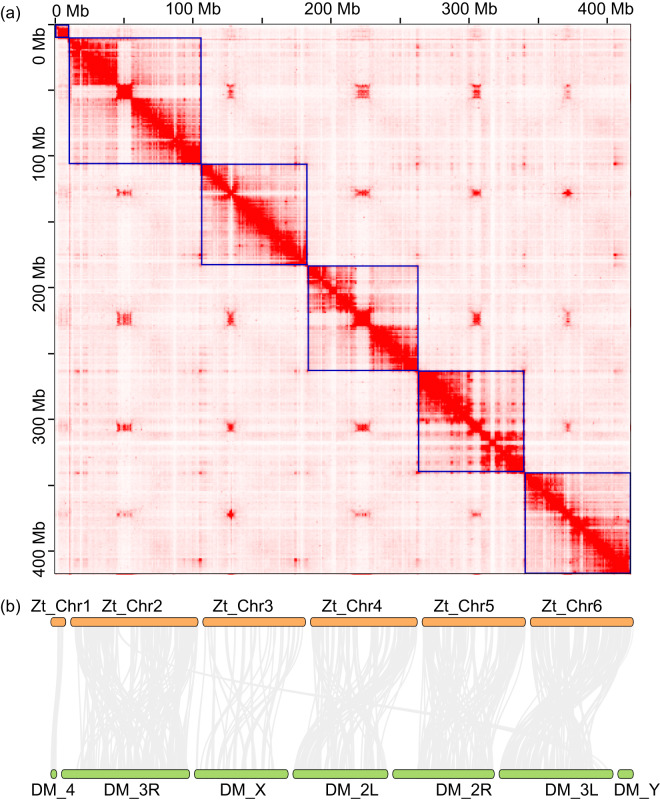
Genome-wide all-by-all Hi-C interaction identified six pseudo-chromosome linkage groups of *Zeugodacus tau* (Zt) genome (**a**) and its synteny between *Drosophila melanogaster* (Dm) genome (**b**).

The *Z. tau* genome has a G + C content of approximately 35.54% (Table 2). At the contig level, we assembled the *Z. tau* genome into 424.74 Mb, consisting of 231 contigs. The contig N50 is 4.23 Mb (Table 2). These contigs were assembled into 421.79 Mb at the chromosomal level, with a scaffold N50 of 77.26 Mb. The chromosome level assembly includes six scaffold groups, with the longest group being 80.04 Mb and the shortest group being 10.74 Mb (Table 2, Fig. 2a). Synteny analysis reveals a highly conserved gene order with small-scale rearrangements and translocations between *Z. tau* and *Drosophila melanogaster* (Fig. 2b). The karyotype of *Z. tau* is 2n = 12, consisting of one pair of heteromorphic sex chromosomes (XX in females, XY in males) and five pairs of autosomes^16,17^. Zt_Chr3 is potentially the X chromosome, showing conserved synteny with the X chromosome of *D. melanogaster*^18^ (Fig. 2b).

**Table 2:** Features of *Zeugodacus* *tau* genome.

Genome features	Values (bp)
Total length (bp)	421,793,426
Contigs N50 (bp)	4,231,206
Scaffold N50 (bp)	77,263,029
Longest scaffold length (bp)	80,036,893
G + C (%)	35.50
Anchored to chromosome (Mb, %)	417,754,029 (99.04%)

### Repeat element and non-coding RNA annotation

Repetitive elements and transposable element families in the assembled genome were detected both by RepeatMasker version 4.0.7^19^ against the Diptera repeats within RepBase Update (http://www.girinst.org) with parameters of ‘-e ABBlast, -species Diptera’. And *ab initio* predicted with the program RepeatModeler version open-1.0.8 (https://www.repeatmasker.org/RepeatModeler/). Most non-coding RNAs (ncRNA) were annotated by aligning the genomic sequence against RFAM (http://rfam.xfam.org/) with version 1.1.2^20^ with the parameter of ‘-e ABBlast’. Transfer RNA (tRNA) was predicted by tRNAscanSE v.1.3.1^21^ with default parameters, and ribosome RNA (rRNA) was predicted by RNAmmer-1.2^22^ with parameters: ‘-S euk, -multi’.

In the *Z. tau* genome, a total of 55.30 Mb sequences (13.15%) were identified as repetitive elements (Table 3). We predicted 247 rRNAs, 435 tRNAs, 67 small nuclear RNAs, and 829 small RNAs in the *Z. tau* genome based on Rfam databases (Table 4).

**Table 3:** Repeats elements statistics in genomes of *Zeugodacus tau*.

Items	Number of elements	Length occupied (bp)	Percentage of sequence (%)
Retroelements	60152	32276426	7.67
SINEs	26	1439	0.00
LINEs	45833	27468074	6.53
LTR elements	14293	4806913	1.14
DNA transposons	77332	17590412	4.18
Total interspersed repeats	NA	55303083	13.15
Small RNA	829	404113	0.10
Satellites	9	1524	0.00
Simple repeats	224888	11697992	2.78
Low complexity	33983	1735037	0.41

Note: SINEs, short interspersed nuclear elements; LINEs, long interspersed nuclear elements; LTR, long terminal repeat.

**Table 4:** Statistics of non-coding RNAs in *Zeugodacus tau* genome.

Class	Type	Number
rRNA counts	8s_rRNA	97
	28s_rRNA	124
	18s_rRNA	53
	Candidate tRNAs read	1675
Cove stats	Cove-confirmed tRNAs	435
	Bases scanned by Cove	211666
tRNA Count	tRNAs decoding Standard 20 AA	433
	Selenocysteine tRNAs (TCA)	1
	Possible suppressor tRNAs (CTA, TTA)	0
	tRNAs with undetermined/unknown isotypes	0
	Predicted pseudogenes	1
Total tRNAs		435
tRNAs with intron		28

### Gene and functional predictions

Protein-coding genes were annotated using homolog-based, RNA-seq-based, and *ab initio* methods in the Maker genome annotation pipeline version 3.01.04^23^ with three iterations. The transcriptome of *Z. tua* was first assembled using StringTie version 1.3.3b^24^ and PASA version 2.0.2^25^. based on the FASTA files of final chromosome assembly and using the transcriptome sequencing reads as input data with default parameters. Homologous genes of *D. melanogaster*^14^ and the transcripts were used to train *ab initio* predicting models for Augustus version 3.4.0^26^ with default parameters and SNAP version 2006-07-28^27^ with the parameters of ‘-categorize 1000, -export 1000, -plus’. The results were used for the next round of model training and annotation. Three rounds of Maker annotations were conducted. The annotated genes were improved by PASA and then filtered based on gene expression evidence and functional annotation. Genes with fragments per kilobase per million (FPKM) value greater than 0 in any RNA-seq data were retained for further analysis. Functions of protein-coding genes, Gene Ontology (GO), and Kyoto Encyclopedia of Genes and Genomes (KEGG) items were annotated using the eggNOG-Mapper version 2.1.9 in the Expected eggNOG DB version 5.0.2^28^ with the parameters of ‘--tax_scope auto, --go_evidence experimental, --target_orthologs all, --seed_ortholog_evalue 0.001, --seed_ortholog_score 60 --override’. A total of 20922 protein-coding genes were annotated in the chromosome-level assembly, in which 17251 genes (82.45%) were functionally annotated.

## Data Records

The *Z. tau* genome project was deposited at NCBI under BioProject No. PRJNA843881^29^. Genomic Nanopore sequencing data were deposited in the Sequence Read Archive at NCBI under accession number SRR19536918^30^. Genomic Illumina sequencing data were deposited in the Sequence Read Archive at NCBI under accession SRR26107452^31^. Hi-C sequencing data were deposited in the Sequence Read Archive at NCBI under accession number SRR26105952^32^. RNA-seq data were deposited in the Sequence Read Archive at NCBI under accession number SRR26086842-SRR26086856^33–46^. The final chromosome assembly was deposited in GenBank at NCBI under accession number GCA_031772095.1^47^. The genome annotation files are available in Figshare under a DOI number of 10.6084/m9.figshare.c.6843474.v2^48^.

## Technical Validation

We evaluated the accuracy of the final genome assembly by aligning Illumina short reads to the *Z. tau* genome using BWA-MEM version 0.7.1721 (https://github.com/lh3/bwa). The analysis revealed that 98.73% of the short reads were successfully mapped to the *Z. tau* genome.

To assess the completeness of the *Z. tau* genome, we conducted analysis using BUSCO version 5.2.2^49^ with the insecta-odb10 database, which consists of 1,367 genes. The BUSCO analysis showed that for the contig level and chromosome level assemblies, 99.7% of the evaluated single-copy genes were identified as complete (single-copied gene: 99.1%, duplicated gene: 0.6%). Additionally, for all protein-coding genes and functionally annotated protein-coding genes, 97.6% (single-copied gene: 96.9%, duplicated gene: 0.7%) and 97.5% (single-copied gene: 96.8%, duplicated gene: 0.7%) were identified as complete, respectively (Table 5).

**Table 5:** Completeness of the assembled genomes and sets of protein-coding genes evaluated by BUSCO analysis.

Data	Complete gene%	Single-copied gene%	Duplicated gene %	Fragmented gene%	Missing gene%
Contig level assembly	99.7	99.1	0.6	0.0	0.3
Chromosome level assembly	99.7	99.3	0.4	0.0	0.3
All protein-coding gene	97.6	96.9	0.7	0.9	1.5
Functionally annotated protein-coding gene	97.5	96.8	0.7	0.9	1.6

## Usage Notes

All data analyses were conducted following the manual and protocols of the published bioinformatic tools. The version and parameters of the software have been described in the Methods section.

## Data Availability

No custom scripts or code were used in this study.
